# Gut microbiota–mediated cardiovascular effects of *Gastrodia elata* polysaccharides: resolving the bioavailability–efficacy paradox

**DOI:** 10.3389/fmicb.2026.1868003

**Published:** 2026-06-10

**Authors:** Ziyi Luo, Shunyao Zhu, Yiqing Lu, Wu Yili, Jiadong Xu

**Affiliations:** 1 Department of Pharmacy, Sir Run Run Shaw Hospital, School of Medicine, Zhejiang University, Hangzhou, Zhejiang, China; 2 School of Pharmaceutical Sciences, Zhejiang Chinese Medical University, Fuchun Campus, Hangzhou, Zhejiang, China; 3 The Second Hospital of Jiaxing, Jiaxing, Zhejiang, China; 4 Department of Physiology, Zhejiang Chinese Medical University, Zhejiang, Hangzhou, China

**Keywords:** cardiovascular disease, *Gastrodia elata*, gut microbiota, gut–heart axis, microbiome, polysaccharides, short-chain fatty acids

## Abstract

**Background:**

Growing evidence suggests that many plant-derived polysaccharides exert systemic effects through gut microbiota–mediated mechanisms rather than direct absorption. *Gastrodia elata* polysaccharides (GEPs) represent a promising but mechanistically complex class of bioactive compounds with potential cardiovascular relevance.

**Objective:**

This review aims to examine the role of gut microbiota in mediating the biological effects of GEPs, with particular focus on resolving the bioavailability–efficacy paradox through host–microbe interactions.

**Methods:**

A narrative synthesis of recent literature was conducted, integrating data on microbiota–polysaccharide interactions, microbial fermentation processes, metabolite production, and downstream host signaling pathways.

**Results:**

Due to limited systemic bioavailability, GEPs undergo extensive fermentation by gut microbiota, generating bioactive metabolites such as short-chain fatty acids and secondary bile acids. These metabolites modulate key host pathways including inflammation, oxidative stress, endothelial function, and lipid metabolism. Emerging evidence highlights the central role of the gut–heart axis in mediating these effects.

**Conclusion:**

The biological activity of GEPs is best understood within a microbiota-centered framework. This perspective provides new insights into polysaccharide pharmacology and supports the development of microbiome-targeted therapeutic strategies.

## 1 Introduction

Cardiovascular disease (CVD) remains the leading cause of death and disability worldwide and continues to impose a substantial clinical and socioeconomic burden across high-, middle-, and low-income settings (Mensah et al., 2023; Vaduganathan et al., 2022). Although major advances in prevention, revascularization, lipid lowering, antithrombotic therapy, and heart failure management have improved survival, the overall burden of CVD continues to rise because of population ageing, persistent exposure to cardiometabolic risk factors, and increasing prevalence of chronic inflammatory and metabolic disorders (Savarese et al., 2023). This evolving landscape highlights the need not only for more effective treatment strategies, but also for adjunctive approaches that target mechanisms incompletely addressed by current therapies.

Despite the success of established cardiovascular drugs, several limitations remain. Residual atherosclerotic risk persists even in patients receiving contemporary lipid-lowering therapy, and ongoing inflammatory, thrombotic, metabolic, and endothelial dysfunction pathways continue to contribute to adverse cardiovascular events (Gomez-Delgado et al., 2024; Bashir et al., 2024; Punnanithinont et al., 2025). In addition, long-term pharmacotherapy may be constrained by incomplete response, intolerance, adverse effects, therapeutic inertia, cost, and poor adherence in real-world practice (Virani et al., 2018). These limitations have stimulated interest in multi-target agents capable of modulating oxidative stress, inflammation, lipid metabolism, vascular injury, and cardiometabolic homeostasis simultaneously.

Within this context, plant-derived polysaccharides have attracted increasing attention as bioactive macromolecules with potentially relevant cardiovascular effects. A growing body of preclinical evidence suggests that natural polysaccharides may exert antioxidant, anti-inflammatory, lipid-lowering, endothelial-protective, antithrombotic, and microbiota-modulating activities, thereby influencing multiple steps in the pathogenesis of atherosclerosis and related cardiovascular disorders. Their broad biological activity, relative biocompatibility, and multi-pathway mode of action make them attractive candidates for exploratory cardiometabolic drug discovery and functional therapeutic development (Dong et al., 2021; Jiang et al., 2022; Cheong et al., 2022).

Among such candidates, *Gastrodia elata* Blume has emerged as a particularly interesting medicinal plant. Traditionally used in East Asian medicine, *G. elata* has been investigated for a wide spectrum of pharmacological effects, including neuroprotective, anti-inflammatory, antioxidant, vasomodulatory, and metabolic actions (Zhu et al., 2019; Yang et al., 2024; Gong et al., 2024). Recent reviews have highlighted the structural diversity and biological relevance of *G. elata* polysaccharides (GEPs), while broader pharmacological syntheses have also suggested possible cardiovascular benefits of this plant and its constituents. Moreover, a dedicated cardiovascular review of *G. elata* emphasized its potential actions on oxidative stress, apoptosis, inflammation, autophagy, vascular dysfunction, and metabolic regulation, all of which are highly relevant to CVD pathobiology (Sun et al., 2023). However, much of the currently available literature remains fragmented across phytochemical, pharmacological, and disease-specific domains. Inference from the recent literature suggests that a focused review centered specifically on GEPs in cardiovascular disease, with emphasis on mechanisms, translational barriers, and future therapeutic positioning, is still insufficiently developed (Yang et al., 2025).

An emerging paradigm in polysaccharide pharmacology highlights the central role of gut microbiota in mediating systemic biological effects. Due to their high molecular weight and limited intestinal absorption, many plant-derived polysaccharides function primarily as substrates for microbial fermentation rather than as directly bioavailable compounds. This process results in the generation of bioactive metabolites, including short-chain fatty acids and other microbial products, which can influence host metabolic, inflammatory, and vascular pathways. Within this context, understanding the gut–heart axis is essential for interpreting the cardiovascular effects of *Gastrodia elata* polysaccharides.

Despite growing interest in plant-derived polysaccharides, the mechanisms underlying their biological effects remain incompletely understood, particularly in the context of cardiovascular disease. A key unresolved issue is the apparent mismatch between strong experimental efficacy and limited systemic bioavailability of these macromolecules. This observation raises the possibility that conventional pharmacological models may not adequately explain their mode of action. In this context, the present review adopts a microbiota-centered perspective and proposes that the cardiovascular effects of GEPs may be substantially influenced by gut microbiota–dependent metabolic processes rather than solely by direct systemic activity of intact polysaccharides. By integrating structural characteristics, pharmacokinetic constraints, and host–microbiome interactions, this review aims to provide a more coherent framework for understanding their therapeutic potential. Importantly, much of the mechanistic framework discussed in this review is derived from indirect evidence, extrapolation from related plant polysaccharides, or broader microbiome literature, while direct mechanistic studies specifically evaluating GEPs remain limited.

**Figure 1:**
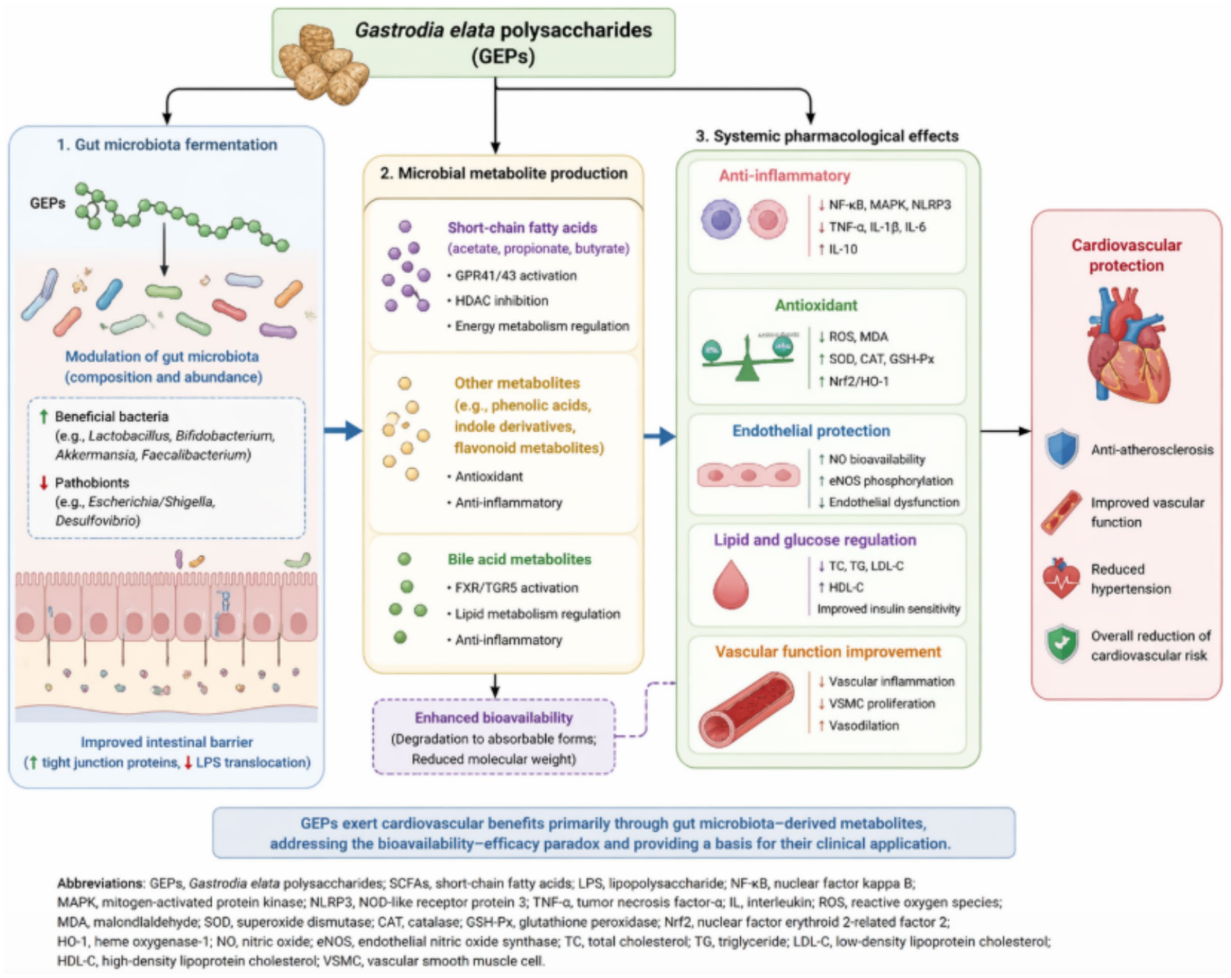
Microbiota-mediated mechanisms underlying the systemic effects of *Gastrodia elata* polysaccharides.

The proposed microbiota-mediated framework underlying the cardiovascular effects of GEPs and the associated bioavailability–efficacy paradox is illustrated in Figure 1.

Current evidence suggests that GEPs may exert cardiovascular protective effects partly through indirect mechanisms involving gut microbiota modulation. Due to their limited systemic bioavailability, GEPs undergo microbial fermentation in the gut, leading to the production of bioactive metabolites such as short-chain fatty acids, bile acid derivatives, and other microbial products. These metabolites influence multiple host signaling pathways, including anti-inflammatory (e.g., NF-κB inhibition), antioxidant (e.g., Nrf2 activation), endothelial function, and lipid metabolism. Collectively, these pathways contribute to improved vascular function and reduced cardiovascular risk. This framework highlights the bioavailability–efficacy paradox and supports a microbiota-centered interpretation of GEP pharmacological activity.

## 2 Chemical composition and structural characteristics of *Gastrodia elata* polysaccharides

Polysaccharides isolated from *Gastrodia elata* (GEPs) represent structurally diverse macromolecules whose biological activity is closely linked to their extraction conditions, molecular architecture, and physicochemical properties. Increasing attention has been directed toward understanding how these structural features influence their pharmacological behavior and biological activity, including mechanisms potentially relevant to cardiovascular protection (Schepetkin and Quinn, 2006; Ferreira et al., 2015). Although substantial progress has been made in characterizing the structural features of GEPs, the direct correlation between these properties and clinically relevant cardiovascular outcomes remains uncertain. This suggests that structural complexity alone may not sufficiently account for their biological effects *in vivo* and highlights the need to consider additional layers of regulation beyond conventional structure–activity relationships.

### 2.1 Extraction and isolation methods

The yield, purity, and structural integrity of GEPs are highly dependent on extraction techniques. Conventional extraction approaches, including hot water extraction and related extraction-assisted methods, are commonly used for polysaccharide isolation because they help preserve polysaccharide integrity; however, these approaches may still result in heterogeneous fractions with variable molecular weights (Chen et al., 2019). To improve extraction efficiency and selectivity, enzyme-assisted extraction (EAE) has been increasingly employed, enabling targeted degradation of plant cell walls and enhancing polysaccharide release without excessive structural disruption (Xu et al., 2019).

More recently, ultrasound-assisted extraction (UAE) and microwave-assisted extraction (MAE) have gained interest as advanced techniques that shorten extraction time, increase yield, and improve reproducibility by enhancing mass transfer and cell wall disruption (Wang et al., 2016). Following extraction, purification steps such as ethanol precipitation, dialysis, and chromatographic fractionation (e.g., DEAE-cellulose and gel filtration chromatography) are typically applied to obtain structurally defined polysaccharide fractions.

Importantly, variations in extraction protocols can significantly alter downstream biological activity, underscoring the need for standardized extraction frameworks in translational research.

### 2.2 Molecular weight distribution and structural heterogeneity

GEPs exhibit considerable structural heterogeneity, including variability in molecular weight and conformational properties, which may influence their biological activity and interactions with host signaling pathways (Leung et al., 2006). This heterogeneity reflects differences in polymer length, branching degree, and conformational complexity.

Emerging evidence suggests that structural characteristics, including molecular weight and conformational complexity, may influence the biological activity and immunomodulatory properties of polysaccharides (Wasser, 2011). In the context of cardiovascular disease, this variability may influence interactions with endothelial cells, immune pathways, and oxidative stress responses, although direct comparative data remain limited.

### 2.3 Monosaccharide composition and glycosidic linkages

Structural analyses indicate that GEPs possess complex polysaccharide architectures composed of multiple monosaccharide residues, which may contribute to their diverse biological and immunomodulatory properties (Ooi and Liu, 2000). The presence and relative proportion of these monosaccharides can vary substantially between studies, reflecting differences in plant origin, extraction conditions, and analytical methods.

Glycosidic linkage analysis indicates that GEPs often contain *β*-(1 → 3), β-(1 → 4), and *α*-(1 → 6) linkages, contributing to a branched and complex three-dimensional architecture. These structural features are critical, as branching patterns and linkage types influence solubility, viscosity, and biological interactions, particularly with cellular receptors and signaling pathways.

### 2.4 Structural features and conformation

Beyond primary composition, higher-order structural characteristics such as branching degree, chain flexibility, and triple-helix conformation have been identified as important determinants of biological activity (Zhang et al., 2005). Advanced analytical techniques including nuclear magnetic resonance (NMR), Fourier-transform infrared spectroscopy (FTIR), and size-exclusion chromatography have enabled more detailed characterization of these conformational features.

Polysaccharides with higher branching and ordered conformations may exhibit enhanced interaction with immune receptors, while more linear or lower molecular weight structures may facilitate diffusion and absorption. However, the precise structural determinants responsible for cardiovascular effects remain incompletely defined, highlighting an important area for future research.

### 2.5 Structure–activity relationships in cardiovascular context

Although direct structure–activity relationship (SAR) studies specific to cardiovascular outcomes are still limited, emerging data suggest that antioxidant, anti-inflammatory, and endothelial-protective effects of GEPs are closely linked to their structural characteristics. For instance, polysaccharides with higher uronic acid content and specific glycosidic linkages have been associated with enhanced free radical scavenging and modulation of inflammatory signaling pathways.

Nevertheless, the lack of standardized characterization across studies presents a major barrier to establishing definitive SAR correlations. Differences in extraction methods, molecular weight distribution, and compositional profiling make it difficult to directly compare results and translate findings into clinically relevant formulations.

## 3 Pharmacokinetics and bioavailability of *Gastrodia elata* polysaccharides

Understanding the pharmacokinetic behavior of *Gastrodia elata* polysaccharides (GEPs) is essential for evaluating their therapeutic potential in cardiovascular disease. Unlike small-molecule drugs, polysaccharides exhibit complex absorption, distribution, metabolism, and excretion (ADME) profiles that are influenced by molecular size, structural conformation, and interactions with the gastrointestinal environment. These unique properties contribute to both their biological effects and their translational limitations (Yu et al., 2018). Importantly, direct pharmacokinetic studies specifically evaluating purified *Gastrodia elata* polysaccharides (GEPs) remain limited. Consequently, several pharmacokinetic concepts discussed in this section are inferred from broader literature involving plant-derived polysaccharides and microbiota-mediated macromolecular metabolism. While these frameworks provide biologically plausible insights into the behavior of GEPs, direct experimental validation specific to GEPs is still insufficient. The limited absorption of high-molecular-weight polysaccharides, coupled with their observed biological activity, indicates a fundamental disconnect between classical pharmacokinetic expectations and experimental outcomes. These observations support the hypothesis of a bioavailability–efficacy paradox, in which indirect mechanisms—particularly those involving gut microbiota metabolism—play a central role in mediating systemic effects. Within this framework, GEPs may act primarily as substrates for microbial fermentation, leading to the generation of bioactive metabolites that influence host signaling pathways relevant to cardiovascular health.

### 3.1 Absorption of polysaccharides

Oral administration is the most common route for polysaccharides; however, their intestinal absorption is inherently limited due to high molecular weight, hydrophilicity, and structural complexity. Most intact polysaccharides are not readily transported across the intestinal epithelium via passive diffusion. Instead, a small fraction may be absorbed through mechanisms such as endocytosis, paracellular transport, or carrier-mediated uptake, although these pathways are generally inefficient and poorly characterized (Schepetkin and Quinn, 2006; De Jesus Raposo et al., 2015).

Recent studies suggest that enzymatic degradation and microbial fermentation of polysaccharides may generate smaller bioactive metabolites capable of influencing host metabolic and signaling pathways (Ríos-Covián et al., 2016), although such observations are derived largely from broader polysaccharide research rather than direct GEP-specific pharmacokinetic studies. Nevertheless, for many plant-derived polysaccharides, including GEPs, systemic bioavailability of the intact macromolecule remains low, indicating that their biological effects may not depend solely on direct absorption into the circulation.

### 3.2 Role of gut microbiota in biotransformation

An emerging concept in polysaccharide pharmacology is the central role of gut microbiota in mediating biological activity. Rather than being absorbed intact, many polysaccharides undergo extensive fermentation by intestinal microbiota, leading to the production of bioactive metabolites such as short-chain fatty acids (SCFAs) and smaller oligosaccharides (Koh et al., 2016; Makki et al., 2018). However, direct characterization of GEP-specific microbial fermentation kinetics and metabolite profiles remains limited.

These microbial metabolites can exert systemic effects through modulation of inflammatory and metabolic pathways, including mechanisms related to glucose homeostasis and host immune regulation that are highly relevant to cardiovascular disease (Agus et al., 2016). In addition, polysaccharides themselves may act as prebiotics, selectively promoting the growth of beneficial microbial populations, thereby indirectly influencing host metabolic and immune responses.

This microbiota-mediated mechanism suggests that the cardiovascular benefits attributed to GEPs may be partly indirect, arising from host–microbiome interactions rather than direct pharmacological action of the intact polysaccharide. Nevertheless, direct studies specifically characterizing microbiota-mediated metabolism of purified GEPs remain limited, and much of the current understanding is extrapolated from broader polysaccharide fermentation literature.

#### 3.2.1 Microbial fermentation pathways and key taxa

Based on broader polysaccharide fermentation literature, gut microbial metabolism is believed to involve a complex community of microorganisms, primarily belonging to the phyla Firmicutes and Bacteroidetes. These proposed microbial interactions remain incompletely validated for purified GEP fractions specifically. These microbes possess specialized carbohydrate-active enzymes (CAZymes) that enable degradation of complex polysaccharides into fermentable substrates. *Bacteroides* species, in particular, are known for their ability to metabolize diverse plant polysaccharides, while members of the Firmicutes phylum contribute significantly to short-chain fatty acid production.

Through these fermentation processes, polysaccharides such as GEPs are converted into metabolites including acetate, propionate, and butyrate. These metabolites play a critical role in host physiology by modulating immune responses, maintaining intestinal barrier integrity, and influencing systemic metabolic pathways.

Variability in gut microbiota composition across individuals may contribute to differences in metabolic response to polysaccharide intake, representing an important consideration for translational application.

### 3.3 Distribution and systemic effects

Following limited absorption or microbial transformation, polysaccharide-derived metabolites can enter the systemic circulation and distribute to target tissues. Although detailed pharmacokinetic data specific to GEPs remain scarce, studies on similar plant polysaccharides indicate that distribution is often non-specific and influenced by molecular size and charge (Yu et al., 2018).

Studies involving plant-derived polysaccharides have reported accumulation in organs such as the liver, spleen, and intestinal mucosa, reflecting the involvement of the reticuloendothelial system and immune-related tissues. However, detailed tissue distribution studies specific to purified GEPs remain scarce. Importantly, systemic biological effects may arise not only from circulating metabolites but also from gut-derived signaling pathways, including immune modulation and enteroendocrine signaling, which can impact distant organs such as the cardiovascular system. However, direct pharmacokinetic and mechanistic studies specific to GEP-derived metabolites remain insufficient.

### 3.4 Metabolism and excretion

Metabolic processing of polysaccharides is primarily driven by microbial enzymatic activity in the colon, rather than hepatic metabolism typical of small molecules, although direct metabolic tracing studies specific to GEPs remain limited (Flint et al., 2012). The resulting metabolites, including SCFAs and monosaccharide derivatives, can be absorbed and further utilized in host metabolic pathways. Excretion pathways include fecal elimination of unabsorbed polysaccharides and renal excretion of low-molecular-weight metabolites. However, the precise metabolic fate of GEPs remains incompletely characterized, and there is a lack of standardized pharmacokinetic profiling across studies.

### 3.5 Challenges: low bioavailability and translational implications

One of the most important limitations affecting the therapeutic development of *Gastrodia elata* polysaccharides (GEPs) is their inherently low and variable systemic bioavailability. Unlike conventional small-molecule drugs, polysaccharides possess high molecular weight, strong hydrophilicity, complex branching structures, and limited membrane permeability, all of which substantially restrict passive diffusion across the intestinal epithelium. In addition, their structural heterogeneity and susceptibility to gastrointestinal degradation further complicate predictable absorption kinetics. As a result, only a limited fraction of intact polysaccharides is believed to reach the systemic circulation following oral administration, creating a discrepancy between measurable plasma exposure and the significant biological effects observed in experimental models.

This apparent mismatch has contributed to the concept of the “bioavailability–efficacy paradox,” in which the therapeutic activity of polysaccharides cannot be adequately explained using traditional pharmacokinetic assumptions alone. Increasing evidence suggests that many of the systemic effects attributed to GEPs may instead arise indirectly through gut microbiota–mediated metabolism. Following intestinal fermentation, GEPs are converted into smaller bioactive metabolites, including short-chain fatty acids, oligosaccharides, and secondary microbial products capable of modulating host inflammatory, metabolic, endothelial, and oxidative stress pathways. Consequently, biological activity may depend less on systemic exposure to intact polysaccharides and more on the dynamic interactions between polysaccharides, gut microbiota composition, and downstream host signaling networks.

Importantly, dependence on microbiota-mediated biotransformation introduces substantial inter-individual variability in therapeutic response. Differences in microbial composition, dietary habits, age, metabolic disease status, antibiotic exposure, and geographic factors may significantly influence fermentation efficiency and metabolite generation. This variability presents a major challenge for dose standardization, reproducibility, and clinical translation, as similar oral doses may produce markedly different biological responses across individuals. Furthermore, many preclinical studies fail to integrate microbiota composition or pharmacokinetic profiling into study design, limiting mechanistic interpretation and reducing translational reliability.

To overcome these limitations, several strategies have been proposed to improve the pharmacological and translational profile of GEPs. Structural modification approaches, including enzymatic hydrolysis and controlled depolymerization, may enhance intestinal permeability and improve metabolic accessibility. Advanced drug delivery systems such as nanoparticles, liposomes, hydrogels, and polysaccharide-based nanocarriers have also been investigated to improve stability, controlled release, and tissue targeting (Torchilin, 2012). In parallel, integration of microbiome profiling, metabolomics, and physiologically based pharmacokinetic (PBPK) modeling may help establish more individualized therapeutic frameworks and improve understanding of host–microbiota interactions underlying GEP activity. Collectively, these approaches may facilitate the development of more standardized and clinically translatable GEP-based interventions. Overall, the pharmacokinetic behavior of GEPs should currently be interpreted as a developing conceptual framework rather than a fully established mechanistic model, highlighting the need for dedicated GEP-specific ADME and metabolite-tracing studies.

## 4 Molecular mechanisms of cardiovascular protection

Many of these molecular pathways may be influenced indirectly through microbiota-derived metabolites, highlighting the importance of host–microbe interactions in mediating the observed biological effects. The cardioprotective potential of *Gastrodia elata* polysaccharides (GEPs) appears to arise from their ability to modulate multiple interconnected biological pathways implicated in the pathogenesis of cardiovascular disease. Rather than acting through a single molecular target, GEPs exert pleiotropic effects involving oxidative stress regulation, inflammatory signaling, endothelial function, lipid metabolism, thrombosis, and myocardial survival pathways. This multi-target profile is particularly relevant given the complex and multifactorial nature of cardiovascular disorders (Madamanchi and Runge, 2019; Libby, 2021). Importantly, although several mechanistic pathways discussed in this section are supported by experimental findings involving GEPs or *Gastrodia elata* extracts, a substantial portion of the proposed signaling framework is also derived from broader literature on plant-derived polysaccharides and microbiota-mediated cardiovascular regulation. Therefore, some mechanistic interpretations should be considered hypothesis-generating and require further validation in GEP-specific experimental systems.

Notably, while many of the described pathways are shared with other plant-derived polysaccharides, emerging evidence suggests that GEPs may exhibit distinct biological behavior due to their unique structural composition, including specific monosaccharide ratios, branching patterns, and molecular weight distribution. These structural characteristics may influence receptor interactions, redox signaling sensitivity, and microbiota-mediated metabolism. However, direct comparative studies between GEPs and other polysaccharides remain limited, representing an important gap in defining GEP-specific mechanistic signatures. Importantly, the mechanistic evidence discussed throughout this section varies considerably in specificity and experimental support. Some observations are derived directly from studies involving purified GEP fractions or *Gastrodia elata* extracts, whereas other proposed mechanisms are extrapolated from broader literature involving plant-derived polysaccharides, microbiota-mediated metabolism, and cardiovascular signaling pathways. Therefore, distinctions between GEP-specific findings and class-wide polysaccharide effects should be interpreted cautiously.

### 4.1 Redox modulation and Nrf2 signaling

Oxidative stress plays a central role in endothelial dysfunction, atherosclerosis progression, and myocardial injury. Excessive production of reactive oxygen species (ROS) contributes to lipid peroxidation, mitochondrial dysfunction, and activation of pro-inflammatory cascades (Madamanchi et al., 2005).

GEPs and related plant-derived polysaccharides have been associated with antioxidant activity and modulation of endogenous antioxidant defense systems, including superoxide dismutase (SOD), catalase, and glutathione peroxidase (Förstermann, 2008). Mechanistically, these effects have been proposed to involve activation of the nuclear factor erythroid 2–related factor 2 (Nrf2) pathway, a key regulator of cellular antioxidant responses (Ma, 2013). However, direct mechanistic validation of Nrf2 pathway activation specifically by purified GEP fractions remains limited.

By reducing oxidative stress burden, GEPs may preserve endothelial integrity, limit oxidative modification of lipoproteins, and attenuate myocardial injury, thereby contributing to cardiovascular protection.

### 4.2 Inflammatory pathway suppression (NF-κB axis)

Chronic low-grade inflammation is a major driver of atherosclerosis and cardiovascular remodeling. Activation of inflammatory pathways, particularly nuclear factor kappa B (NF-κB) signaling, leads to increased expression of cytokines, adhesion molecules, and pro-thrombotic mediators (Hansson, 2005). Evidence from studies involving GEPs and related natural polysaccharides suggests potential suppression of inflammatory signaling by inhibiting NF-κB activation and reducing the production of pro-inflammatory cytokines such as tumor necrosis factor-α (TNF-α) and interleukin-6 (IL-6), although much of the mechanistic interpretation is extrapolated from broader polysaccharide and inflammatory pathway literature (Förstermann, 2008; Moore and Tabas, 2011). Notably, several mechanistic interpretations regarding NF-κB modulation are extrapolated from broader polysaccharide literature rather than direct GEP-specific experimental studies. Additionally, modulation of macrophage polarization toward an anti-inflammatory phenotype has been proposed as a contributing mechanism (Moore and Tabas, 2011). These anti-inflammatory effects are particularly relevant in the context of plaque stability and prevention of vascular injury progression.

### 4.3 Endothelial signaling and NO bioavailability

Endothelial dysfunction is a fundamental early event in the development and progression of cardiovascular disease and plays a central role in atherosclerosis, hypertension, thrombosis, and vascular inflammation. A key feature of endothelial dysfunction is the impairment of nitric oxide (NO) bioavailability, resulting from reduced endothelial nitric oxide synthase (eNOS) activity, excessive oxidative stress, and chronic inflammatory signaling. Under pathological conditions, increased production of reactive oxygen species (ROS) promotes oxidative degradation of NO, leading to impaired vasodilation, endothelial activation, leukocyte adhesion, and vascular remodeling.

Emerging evidence suggests that *Gastrodia elata* polysaccharides (GEPs) may improve endothelial function through multiple interconnected mechanisms. One proposed mechanism involves enhancement of eNOS activity and preservation of NO signaling pathways, thereby improving endothelial-dependent vasodilation and vascular homeostasis. Experimental and mechanistic studies suggest that activation of endothelial survival and signaling pathways, including phosphatidylinositol 3-kinase (PI3K)/Akt-mediated eNOS phosphorylation, may contribute to increased NO production and improved endothelial responsiveness (Förstermann and Sessa, 2012; Vanhoutte et al., 2017). Although direct evidence specific to GEPs remains limited, their antioxidant and anti-inflammatory properties strongly support a mechanistic role in endothelial protection.

In addition to promoting NO synthesis, GEPs may preserve NO bioavailability indirectly through attenuation of oxidative stress. Excessive ROS generation not only damages endothelial cells but also rapidly inactivates NO through formation of peroxynitrite, thereby impairing vascular relaxation. Through modulation of antioxidant pathways such as Nrf2 and enhancement of endogenous antioxidant defense systems including superoxide dismutase and glutathione peroxidase, GEPs may potentially reduce oxidative NO degradation and improve endothelial redox balance (Förstermann, 2008; Ma, 2013). This reduction in oxidative stress may subsequently improve vascular compliance and reduce progression of endothelial injury.

Inflammatory signaling pathways also play a major role in endothelial dysfunction. Activation of NF-κB promotes expression of adhesion molecules including vascular cell adhesion molecule-1 (VCAM-1) and intercellular adhesion molecule-1 (ICAM-1), facilitating leukocyte recruitment and vascular inflammation (Hansson, 2005). Through suppression of NF-κB-mediated inflammatory signaling and reduction of pro-inflammatory cytokines such as TNF-α and IL-6, GEPs may attenuate endothelial activation and reduce vascular inflammatory burden (Moore and Tabas, 2011). Furthermore, microbiota-derived metabolites generated following GEP fermentation, particularly short-chain fatty acids, may additionally contribute to endothelial protection through modulation of immune signaling and maintenance of vascular homeostasis.

Collectively, these mechanisms suggest that the endothelial-protective effects of GEPs arise from coordinated regulation of oxidative stress, inflammatory signaling, NO bioavailability, and microbiota-mediated metabolic pathways rather than from a single molecular target alone.

### 4.4 Lipid regulatory pathways and PPAR-mediated signaling

Dysregulation of lipid metabolism is a major contributor to atherosclerosis and cardiovascular disease progression. Beyond elevated low-density lipoprotein (LDL) levels, abnormalities in fatty acid oxidation, lipid accumulation, endothelial lipid toxicity, and chronic metabolic inflammation collectively contribute to vascular injury and plaque formation. In this context, peroxisome proliferator-activated receptors (PPARs) represent key transcriptional regulators linking lipid metabolism, inflammation, and cardiometabolic homeostasis (Staels and Fruchart, 2005; Ferré, 2004).

Emerging evidence suggests that the lipid-modulating effects of *Gastrodia elata* polysaccharides (GEPs) may involve activation of multiple PPAR-dependent signaling pathways. Among these, PPAR-*α* is particularly important in regulating hepatic fatty acid β-oxidation, lipoprotein metabolism, and triglyceride clearance. Activation of PPAR-α promotes transcription of genes involved in fatty acid transport and oxidation, including carnitine palmitoyltransferase-1 (CPT-1) and acyl-CoA oxidase, thereby reducing circulating triglyceride levels and limiting lipid accumulation within vascular tissues. Through these mechanisms, GEPs may contribute to improved lipid handling and attenuation of atherosclerotic progression. However, direct experimental studies specifically evaluating PPAR subtype activation by purified GEP fractions remain limited.

PPAR-*γ* signaling may also play an important role in mediating the cardiometabolic effects of GEPs. In addition to regulating adipocyte differentiation and glucose metabolism, PPAR-*γ* exerts significant anti-inflammatory and anti-atherogenic effects within vascular tissues. Activation of PPAR-γ suppresses NF-κB-driven inflammatory signaling, reduces macrophage activation, and inhibits formation of lipid-laden foam cells, which are central to plaque development. Experimental evidence from studies involving plant-derived polysaccharides suggests that modulation of PPAR-*γ* activity may improve insulin sensitivity, reduce oxidative stress, and stabilize vascular inflammatory responses.

Importantly, microbiota-derived metabolites generated following GEP fermentation may further contribute to PPAR activation. Short-chain fatty acids produced through microbial polysaccharide metabolism have been shown to influence lipid and energy homeostasis through interactions with PPAR signaling networks and associated metabolic pathways. This suggests that some lipid-regulatory effects attributed to GEPs may arise indirectly through gut microbiota-mediated metabolic signaling rather than direct activity of intact polysaccharides alone.

Downstream consequences of PPAR activation may include reduced LDL accumulation, improved high-density lipoprotein (HDL) metabolism, suppression of vascular inflammation, attenuation of oxidative stress, and stabilization of atherosclerotic plaques. Collectively, these interconnected pathways position PPAR signaling as a potentially important mechanistic link between GEP-mediated microbiota interactions and cardiovascular protection. Although these mechanisms are biologically plausible, much of the current understanding regarding polysaccharide-mediated PPAR regulation is derived from broader studies involving natural polysaccharides, while direct experimental evidence specific to purified GEP fractions remains comparatively limited.

### 4.5 Anti-thrombotic and anti-platelet effects

Thrombosis plays a central role in acute cardiovascular events such as myocardial infarction and stroke. Platelet activation, aggregation, and coagulation cascade activation are key contributors to thrombus formation (Jackson, 2011).

Experimental and mechanistic studies involving natural bioactive compounds suggest potential anti-platelet and anti-thrombotic effects through mechanisms including inhibition of platelet aggregation, modulation of thromboxane-related pathways, and reduction of endothelial injury-associated pro-thrombotic signaling (Ruggeri, 2002).

### 4.6 Cardiomyocyte protection and anti-apoptotic signaling

Cardiomyocyte injury and apoptosis are central features of ischemia–reperfusion injury and heart failure progression. Cellular apoptosis is regulated by multiple pathways, including mitochondrial dysfunction and activation of pro-apoptotic signaling cascades (Turer and Hill, 2010).

Experimental observations and broader apoptosis-related literature suggest that GEPs may exert protective effects in myocardial injury models through mechanisms involving reduction of oxidative stress-associated apoptosis, modulation of Bcl-2/Bax signaling balance, and inhibition of caspase activation pathways, thereby potentially contributing to preservation of myocardial structure and function under stress conditions (Elmore, 2007).

**Figure 2:**
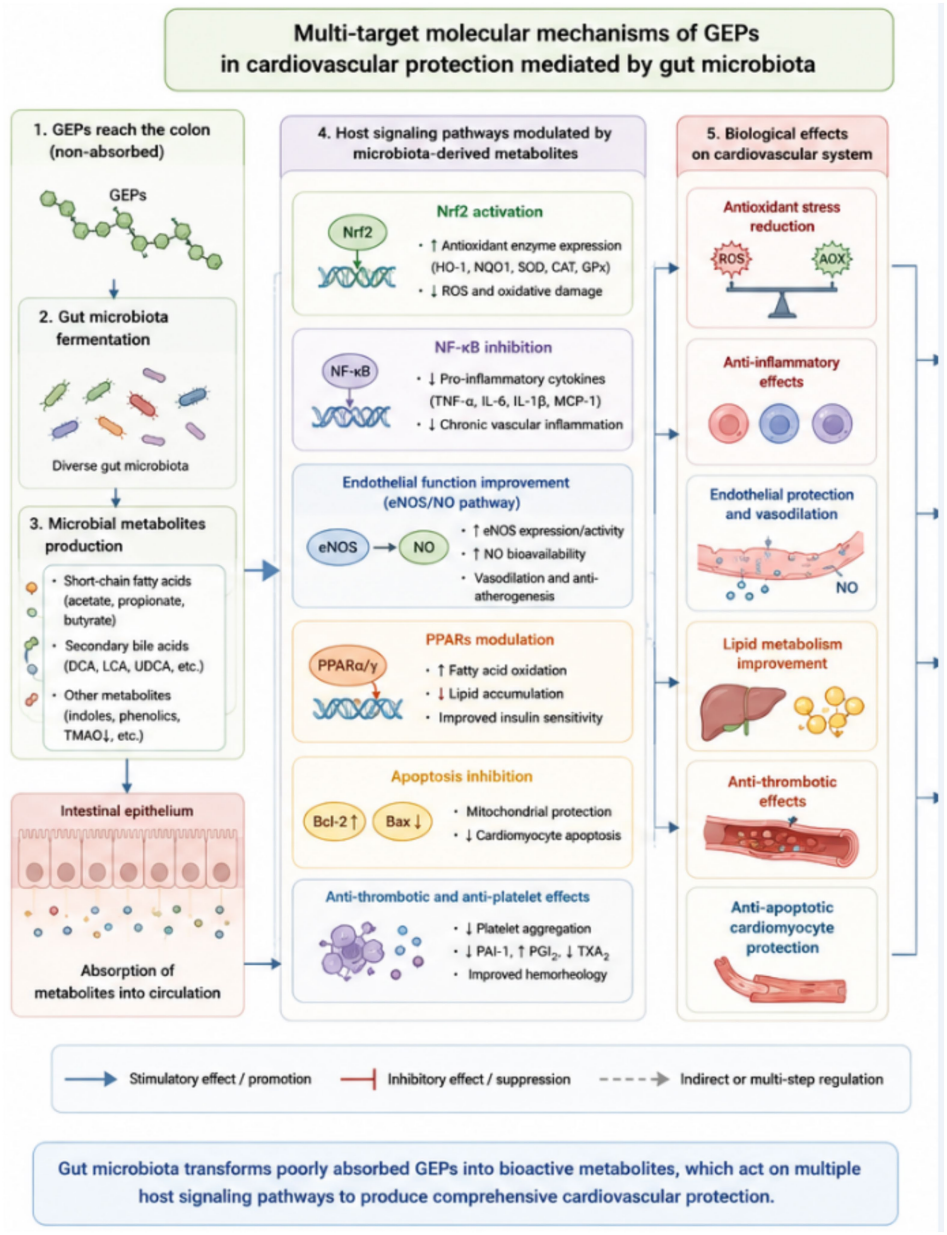
Multi-target molecular mechanisms of GEP-mediated cardiovascular protection through gut microbiota interactions.

The key molecular pathways involved in GEP-mediated cardioprotection are summarized in Table 1. A schematic overview of the microbiota-mediated multi-target molecular mechanisms involved in GEP-induced cardiovascular protection is presented in Figure 2.

**Table 1:** Proposed molecular mechanisms underlying the cardiovascular protective effects of *Gastrodia elata* polysaccharides.

Mechanism	Pathway	Effect	Clinical relevance
Antioxidant	Nrf2	↓ ROS	Atherosclerosis
Anti-inflammatory	NF-κB	↓ TNF-α, IL-6	Plaque stability
Endothelial	eNOS/NO	↑ vasodilation	Hypertension
Lipid metabolism	PPAR	↓ LDL	Atherosclerosis
Anti-thrombotic	Platelet inhibition	↓ aggregation	MI/stroke
Anti-apoptotic	Bcl-2/Bax	↓ cell death	MI/HF

Nrf2, nuclear factor erythroid 2–related factor 2; NF-κB, nuclear factor kappa B; eNOS, endothelial nitric oxide synthase; NO, nitric oxide; PPAR, peroxisome proliferator-activated receptor; LDL, low-density lipoprotein; TNF-α, tumor necrosis factor-alpha; IL-6, interleukin-6; MI, myocardial infarction; HF, heart failure. While these pathways are supported by experimental evidence, most findings are derived from preclinical models, and the relative contribution of each mechanism to clinical cardiovascular outcomes remains uncertain.

Following oral administration, GEPs undergo fermentation by gut microbiota, leading to the generation of bioactive metabolites including short-chain fatty acids and secondary bile acid derivatives. These metabolites are proposed to influence multiple interconnected signaling pathways involved in cardiovascular protection, including activation of Nrf2-mediated antioxidant responses, inhibition of NF-κB-driven inflammation, enhancement of endothelial nitric oxide synthase (eNOS)/nitric oxide signaling, regulation of lipid metabolism through peroxisome proliferator-activated receptors (PPARs), suppression of platelet activation, and modulation of apoptosis-related pathways such as Bcl-2/Bax signaling. Collectively, these mechanisms may contribute to reduced oxidative stress, improved endothelial function, attenuation of vascular inflammation, plaque stabilization, and cardiomyocyte protection. Nevertheless, several proposed signaling pathways remain inferential and require direct validation in GEP-specific cardiovascular and microbiome-integrated experimental models.

Beyond individual molecular pathways, the biological effects of GEPs are increasingly understood within an integrated framework involving limited direct absorption and significant microbiota-mediated metabolism. Following oral administration, GEPs undergo fermentation by gut microbiota, leading to the generation of bioactive metabolites such as short-chain fatty acids, which exert systemic effects on oxidative stress, inflammation, endothelial function, and lipid metabolism. These interconnected mechanisms collectively contribute to cardiovascular protection (Koh et al., 2016; Makki et al., 2018; Agus et al., 2016; Tang et al., 2019; Witkowski et al., 2020).

Interpreting these molecular pathways within a conventional pharmacological framework may overlook an important dimension of GEP activity. Given their limited systemic bioavailability, it is plausible that many of the observed effects arise from downstream signaling triggered by microbiota-derived metabolites rather than direct interaction of intact polysaccharides with target tissues. This perspective positions GEPs as upstream modulators of complex biological networks rather than direct receptor-targeting agents.

## 5 Evidence from preclinical and clinical studies

Current understanding of the cardiovascular effects of GEPs is derived predominantly from mechanistic, *in vitro*, and animal-based studies exploring inflammatory, oxidative, and vascular pathways relevant to atherosclerosis and cardiovascular injury. Although these findings suggest potential multi-target cardioprotective properties, direct clinical validation remains limited, and the translational strength of the available evidence is constrained by methodological heterogeneity and the scarcity of well-designed human studies (Libby, 2002; Hansson, 2005). The predominance of preclinical evidence must also be interpreted with caution, as experimental conditions may not fully capture the complexity of host–microbiome interactions present in human systems. As a result, the magnitude and mechanisms of observed effects in controlled models may not directly translate to clinical settings, reinforcing the need for a more integrative interpretation of existing data.

### 5.1 *In vitro* studies

Cell-based and mechanistic studies have provided preliminary insights into pathways potentially relevant to the biological activity of GEPs, particularly those involving oxidative stress, inflammation, and endothelial dysfunction. Experimental evidence from vascular and oxidative stress models suggests that modulation of reactive oxygen species (ROS) generation and inflammatory signaling pathways may contribute to improved cellular resilience under stress conditions (Cai and Harrison, 2000).

Experimental observations from myocardial injury and endothelial stress models suggest that modulation of nitric oxide signaling, endothelial activation, oxidative stress, apoptosis, and mitochondrial dysfunction may contribute to the potential protective effects attributed to GEPs and related polysaccharides under hypoxic or oxidative stress conditions (Yellon and Hausenloy, 2007).

However, *in vitro* findings must be interpreted cautiously, as experimental systems frequently employ supraphysiological concentrations, simplified cellular environments, and short-term exposure conditions that may not accurately reflect the pharmacokinetic and biological complexity present *in vivo*.

### 5.2 Animal models

Animal and experimental cardiovascular studies provide indirect mechanistic support for pathways potentially relevant to the cardiovascular effects of GEPs, particularly those involving oxidative stress, endothelial dysfunction, vascular inflammation, and atherosclerotic progression. These findings suggest possible interactions with mechanisms related to blood pressure regulation, lipid metabolism, and vascular function, although direct GEP-specific animal evidence remains relatively limited and heterogeneous (Guzik et al., 2000; Glass and Witztum, 2001).

Experimental studies of myocardial ischemia–reperfusion injury suggest that modulation of oxidative stress and apoptotic signaling pathways may contribute to potential cardioprotective effects attributed to GEPs and related polysaccharides, including mechanisms associated with myocardial injury attenuation and preservation of cellular integrity (Hausenloy and Yellon, 2013). Additionally, experimental studies have reported attenuation of vascular inflammatory responses, including reduced expression of pro-inflammatory mediators and preservation of endothelial integrity. Despite these promising observations, important limitations remain, including substantial variability in animal models and disease induction methods, inconsistent extraction procedures and dosing regimens, and lack of standardized outcome measures across studies. These factors complicate direct comparison between studies and limit the translational interpretation of preclinical findings.

Moreover, many animal models incompletely capture the multifactorial nature of human cardiovascular disease, including aging, metabolic comorbidities, polypharmacy, and long-term inflammatory remodeling, thereby limiting translational extrapolation.

### 5.3 Human studies

Clinical evidence evaluating the cardiovascular effects of *Gastrodia* elata or its polysaccharide components remains limited, and important methodological challenges persist in translating experimental findings into clinically robust evidence. Many available studies are characterized by small sample sizes, short durations, heterogeneous interventions, or the use of whole plant extracts rather than purified polysaccharides, making interpretation and generalization difficult (Ford and Norrie, 2016).

Accordingly, the current clinical evidence should be regarded as preliminary and insufficient to establish definitive therapeutic efficacy.

Clinical evidence supporting cardiovascular applications of GEPs remains limited; however, broader cardiovascular prevention studies continue to highlight the importance of lipid regulation and metabolic risk reduction in reducing cardiovascular disease burden (Yusuf et al., 2016).

However, these studies often lack rigorous design elements such as randomization, blinding, and standardized dosing protocols.

Importantly, there is a notable absence of large-scale randomized controlled trials (RCTs) specifically evaluating GEPs in cardiovascular disease. This represents a major gap in the evidence base and limits the ability to draw definitive conclusions regarding efficacy and safety.

### 5.4 Critical appraisal of evidence

While preclinical studies provide a potentially important mechanistic rationale for the cardiovascular effects of GEPs, the overall evidence base remains preliminary and heterogeneous. Current limitations include substantial reliance on *in vitro* and animal data, inadequate integration of pharmacokinetic considerations into experimental design, insufficient standardization of polysaccharide characterization methods, and the scarcity of robust clinical validation studies. Collectively, these limitations constrain mechanistic interpretation, reproducibility, and clinical translation. Moreover, many studies focus on surrogate biomarkers rather than clinically meaningful outcomes such as cardiovascular events or mortality.

Importantly, much of the currently available evidence should be interpreted as exploratory rather than definitive. Many experimental studies utilize non-physiological or supraphysiological dosing regimens that may not accurately reflect clinically achievable exposure levels, particularly given the limited systemic bioavailability of intact polysaccharides. In addition, commonly used rodent and cell-based cardiovascular models incompletely replicate the complexity, chronicity, and comorbidity burden characteristic of human cardiovascular disease. Considerable heterogeneity also exists across studies with respect to extraction methods, polysaccharide characterization, disease models, dosing protocols, treatment duration, and outcome assessment, thereby limiting reproducibility and cross-study comparability. Consequently, while the current evidence provides important mechanistic and hypothesis-generating insights, many reported cardioprotective effects of GEPs should still be considered preliminary pending validation in standardized translational and clinical studies.

A summary of key preclinical and clinical studies is presented in Table 2.

**Table 2:** Representative mechanistic and translational evidence relevant to the cardiovascular effects of *Gastrodia* elata polysaccharides (GEPs).

Study	Model	Intervention/focus	Key findings	Mechanistic relevance
Cai and Harrison (2000)	Endothelial oxidative stress models	Oxidative stress pathways	Reactive oxygen species contribute to endothelial dysfunction and vascular injury	Antioxidant and endothelial-protective mechanisms potentially relevant to GEPs
Yellon and Hausenloy (2007)	Myocardial ischemia–reperfusion models	Myocardial reperfusion injury	Oxidative stress and apoptosis are major mediators of myocardial injury	Cardiomyocyte protection and anti-apoptotic signaling
Guzik et al. (2000)	Vascular dysfunction studies	NAD(P)H oxidase-mediated oxidative stress	Increased vascular superoxide production associated with endothelial dysfunction	Oxidative stress modulation and vascular protection
Glass and Witztum (2001)	Atherosclerosis mechanistic review	Atherosclerotic inflammation and lipid injury	Chronic inflammation and lipid dysregulation drive plaque progression	Lipid metabolism and vascular inflammatory pathways
Hausenloy and Yellon (2013)	Myocardial ischemia–reperfusion review	Cardioprotective therapeutic targets	Modulation of oxidative stress and apoptosis may reduce myocardial injury	Anti-apoptotic and antioxidant cardioprotection
Ford and Norrie (2016)	Clinical trial methodology review	Pragmatic cardiovascular trials	Translation of experimental findings requires robust clinical trial design	Translational and clinical evidence limitations
Yusuf et al. (2016)	Cardiovascular prevention trial	Lipid-lowering intervention	Cardiovascular risk reduction linked to improved metabolic control	Clinical relevance of cardiometabolic risk modulation
Dong et al. (2021)	Review of plant polysaccharides	Plant-derived polysaccharides in cardiovascular disease	Plant polysaccharides exhibit antioxidant, anti-inflammatory, and vascular protective effects	Multi-target cardioprotective mechanisms
Jiang et al. (2022)	Review of bioactive polysaccharides	Anti-atherosclerotic mechanisms	Polysaccharides may improve lipid metabolism and inflammatory regulation	Anti-atherosclerotic and metabolic effects
Tang et al. (2019)	Gut microbiota and cardiovascular disease review	Microbiota-derived metabolites	Microbial metabolites influence inflammation and cardiometabolic homeostasis	Microbiota-mediated cardiovascular mechanisms

GEPs, *Gastrodia elata* polysaccharides; ROS, reactive oxygen species; NO, nitric oxide; LDL, low-density lipoprotein.

Most available evidence remains indirect and is derived predominantly from mechanistic, *in vitro*, animal, or broader polysaccharide literature rather than direct GEP-specific cardiovascular clinical trials. Variability in extraction methods, polysaccharide composition, dosing strategies, and study design should therefore be considered when interpreting translational relevance.

## 6 Translational gaps and limitations

A major limitation in the current literature is the continued reliance on traditional pharmacological frameworks that may not fully account for the unique biological, metabolic, and translational properties of complex polysaccharides and herbal-derived compounds. In particular, insufficient consideration has been given to the possibility that their biological effects are mediated indirectly through microbiota-dependent mechanisms. This conceptual gap contributes to challenges in translating preclinical findings into clinically meaningful outcomes (Efferth and Kaina, 2011; Sonnenburg and Bäckhed, 2016).

### 6.1 Structural heterogeneity and lack of standardization

A fundamental limitation in GEP research is the absence of a standardized molecular entity. Unlike conventional pharmacological agents, GEPs represent heterogeneous mixtures of polysaccharide fractions that vary in molecular weight, monosaccharide composition, branching architecture, and physicochemical characteristics (Tzianabos, 2000). In addition, variability in plant source, cultivation conditions, extraction and purification procedures, and analytical characterization methods contributes substantially to inter-study heterogeneity. These inconsistencies make it difficult to compare experimental findings, establish reproducible therapeutic formulations, or define reliable dose–response relationships. Consequently, the lack of standardization remains a major barrier to mechanistic interpretation, regulatory evaluation, and clinical translation.

### 6.2 Pharmacokinetic uncertainty and microbiota-dependent bioavailability

As discussed previously, GEPs exhibit poor and inconsistent systemic bioavailability, largely due to their macromolecular structure and dependence on gut microbiota-mediated metabolism (Vetvicka and Vetvickova, 2007). Nevertheless, many preclinical investigations inadequately address pharmacokinetic considerations, including absorption behavior, tissue distribution, dose-exposure relationships, and the potential influence of microbiota variability on biological responses. This disconnect between pharmacokinetics and pharmacodynamics represents an important translational limitation, as rational therapeutic dosing strategies cannot be reliably established without a clearer understanding of systemic exposure and metabolic processing.

### 6.3 Over-reliance on preclinical models

The current evidence base is heavily skewed toward *in vitro* and animal studies, which, although valuable for mechanistic exploration, have inherent limitations in predicting human outcomes (van der Worp et al., 2010; Pound et al., 2004). Many experimental models rely on simplified disease conditions that fail to adequately capture the complexity of human cardiovascular disorders, including the influence of aging, diabetes, multimorbidity, and polypharmacy. In addition, the frequent use of supra-physiological dosing regimens and highly controlled laboratory conditions may contribute to overestimation of therapeutic efficacy and limit the external validity and clinical translatability of preclinical findings.

### 6.4 Scarcity of high-quality clinical evidence

Perhaps the most important limitation is the near absence of rigorously designed clinical trials evaluating GEPs in cardiovascular disease (Ridker et al., 2018). Existing human evidence remains limited and methodologically heterogeneous, with many studies relying on short-term interventions, variable formulations, and surrogate biomarkers such as lipid parameters or blood pressure measurements rather than clinically meaningful cardiovascular outcomes. Consequently, the current evidence base remains insufficient to establish definitive therapeutic efficacy, optimal dosing strategies, or long-term clinical applicability.

### 6.5 Insufficient mechanistic depth and lack of systems-level validation

Despite growing interest in the cardiovascular effects of *Gastrodia elata* polysaccharides (GEPs), mechanistic understanding remains incomplete and fragmented. Most currently available studies focus on isolated molecular pathways, such as antioxidant or anti-inflammatory signaling, without adequately integrating these findings into broader systems-level biological networks. As a result, the precise sequence of events linking GEP administration to downstream cardiovascular effects remains insufficiently defined.

An additional limitation is the heavy reliance on associative pathway analyses rather than direct mechanistic validation. Many studies report alterations in biomarkers such as Nrf2, NF-κB, or inflammatory cytokines without establishing causal relationships through pathway inhibition experiments, gene knockdown approaches, receptor validation, or metabolite-tracing methodologies. Consequently, it remains uncertain which molecular pathways are primary drivers of therapeutic activity and which represent secondary downstream responses.

Furthermore, although the gut microbiota is increasingly recognized as a central mediator of GEP activity, direct microbiome-integrated mechanistic studies remain scarce. Few investigations incorporate metagenomics, metabolomics, microbial functional profiling, or host–microbiome interaction analyses capable of clarifying how microbial fermentation products influence cardiovascular signaling pathways. This represents a major conceptual limitation because microbiota-mediated metabolism may constitute the principal mechanism underlying the bioavailability–efficacy paradox discussed throughout this review.

The absence of integrated systems biology approaches also limits translational interpretation. Future studies should incorporate multi-omics methodologies, including transcriptomics, metabolomics, proteomics, and microbiome sequencing, combined with network pharmacology and functional validation strategies. Such approaches may help identify key signaling hubs, define mechanistic hierarchies, and improve understanding of the complex interactions linking GEPs, gut microbiota, and cardiovascular physiology.

### 6.6 Safety, toxicity, and regulatory considerations

Although plant-derived polysaccharides are generally perceived as biologically safe and well tolerated, the long-term safety profile of GEPs remains insufficiently characterized, particularly in relation to chronic administration, cardiovascular polypharmacy, and potential herb–drug interactions (Zhou and Lai, 2008; Izzo and Ernst, 2009).

Most currently available studies primarily focus on efficacy-related outcomes, while systematic toxicological evaluation, chronic exposure assessment, and pharmacovigilance-related investigations remain limited. This imbalance represents an important translational concern, particularly given the potential need for prolonged administration in chronic cardiovascular disease settings.

Several safety-related uncertainties remain unresolved. First, substantial batch-to-batch variability arising from differences in plant source, cultivation conditions, extraction methods, and purification protocols may lead to inconsistent chemical composition and unpredictable biological effects. Second, the microbiota-dependent metabolism of GEPs introduces additional variability in metabolite generation, raising the possibility that individual differences in gut microbial composition may influence not only therapeutic efficacy but also tolerability and off-target biological responses.

Potential interactions with standard cardiovascular medications also remain poorly investigated. Given the reported anti-inflammatory, anti-platelet, and metabolic effects of GEPs, theoretical interactions with antiplatelet agents, anticoagulants, statins, antihypertensive drugs, and glucose-lowering therapies cannot be excluded. However, formal drug–herb interaction studies are currently lacking.

Another important limitation is the scarcity of standardized toxicological studies evaluating hepatic, renal, immunological, and gastrointestinal safety following long-term exposure. Existing evidence is largely derived from short-duration animal studies using heterogeneous formulations, limiting extrapolation to clinical use. In addition, regulatory frameworks for polysaccharide-based therapeutics remain insufficiently developed, particularly regarding quality control, molecular characterization, manufacturing reproducibility, and microbiota-related pharmacodynamic variability.

**Figure 3:**
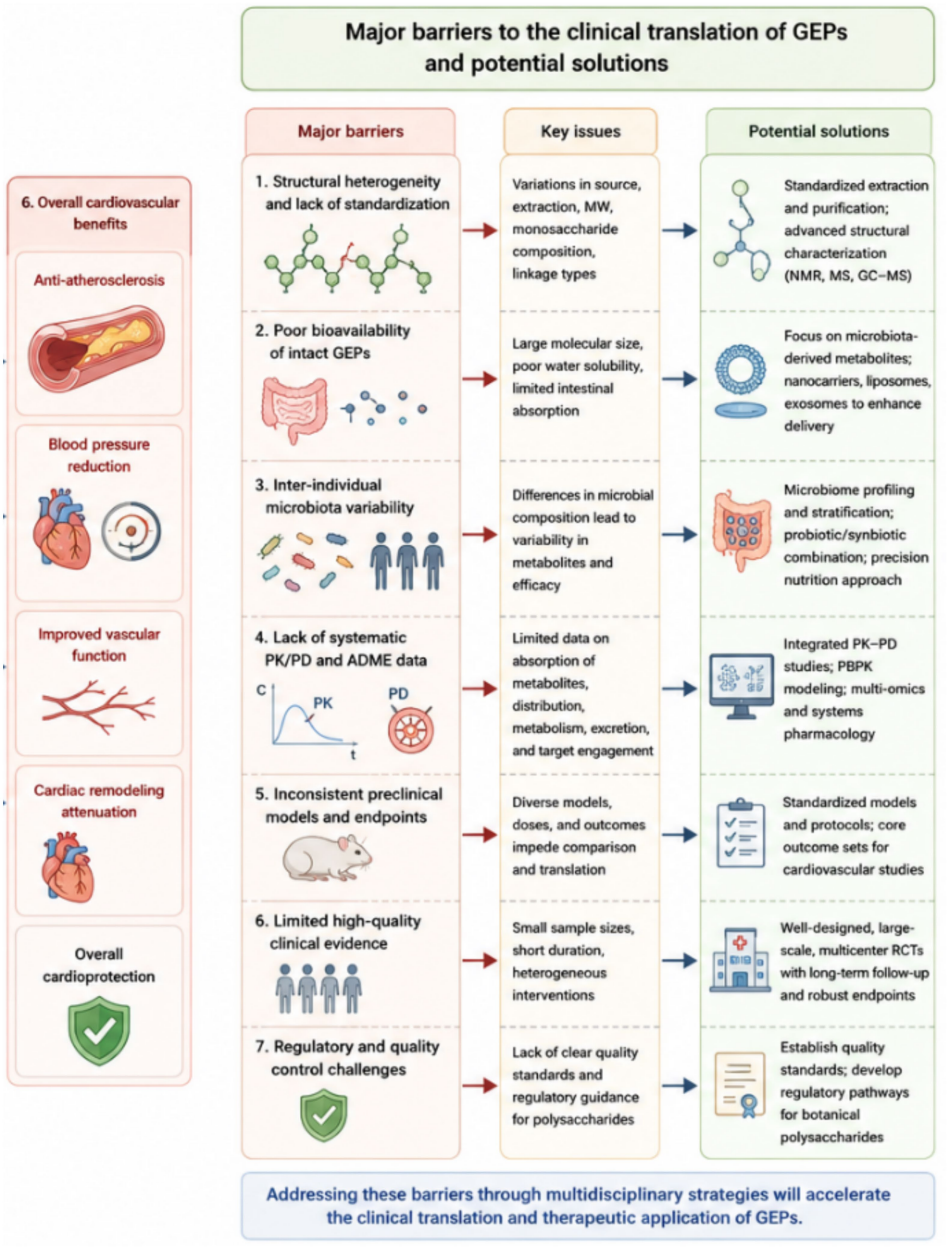
Major barriers to the clinical translation of *Gastrodia elata* polysaccharides (GEPs) and potential solutions.

Collectively, these limitations highlight the need for comprehensive safety assessment strategies incorporating chronic toxicity studies, standardized manufacturing protocols, microbiome-related safety profiling, and rigorous clinical monitoring before widespread cardiovascular application of GEP-based interventions can be considered. The major translational barriers limiting the clinical development of GEPs and the corresponding potential strategies to overcome these challenges are summarized in Figure 3.

The figure summarizes key translational limitations affecting the development of GEP-based cardiovascular therapeutics, including structural heterogeneity, poor bioavailability, microbiota variability, lack of PK–PD integration, limited clinical evidence, and regulatory challenges. Potential solutions include standardized molecular characterization, microbiome-guided therapeutic strategies, advanced delivery systems, multi-omics integration, and large-scale randomized clinical trials.

## 7 Future therapeutic directions

Future research should move beyond reductionist approaches that focus solely on isolated molecular pathways and instead adopt a systems-level perspective integrating pharmacokinetics, microbiota metabolism, and host signaling networks. Such an approach is essential for accurately defining the therapeutic role of GEPs and for designing interventions that align with their underlying mechanisms of action. Future investigations should also incorporate multi-omics approaches, including metabolomics, transcriptomics, microbiome profiling, and systems pharmacology, to better characterize the complex host–microbiota interactions and molecular mechanisms underlying GEP-mediated cardiovascular effects (Hasin et al., 2017).

### 7.1 Standardization and molecular definition of GEPs

A critical priority is the development of standardized extraction, purification, and analytical characterization protocols to improve reproducibility across studies. More detailed structural characterization, including evaluation of molecular weight distribution, monosaccharide composition, branching architecture, and glycosidic linkage patterns, will be important for improving mechanistic understanding and facilitating more consistent interpretation of biological activity (Wasser, 2014; Brown and Gordon, 2005).

Advanced analytical tools, including high-resolution mass spectrometry and nuclear magnetic resonance profiling, should be routinely integrated to define structure–function relationships at a molecular level, enabling the identification of bioactive fractions with consistent therapeutic potential.

### 7.2 Integration of pharmacokinetic-pharmacodynamic (PK-PD) modeling

Future studies should incorporate pharmacokinetic–pharmacodynamic (PK–PD) frameworks to better understand dose–response relationships and optimize therapeutic regimens. Given the complex absorption and microbiota-mediated metabolism of GEPs, traditional pharmacokinetic models may be insufficient.

Innovative approaches, including systems pharmacology and physiologically based pharmacokinetic (PBPK) modeling, may provide important insights into tissue distribution, target engagement, time-dependent biological responses, and inter-individual variability. Integration of these approaches could facilitate translation of preclinical findings into more clinically meaningful and mechanism-informed dosing strategies (Hopkins, 2008; Jones et al., 2015).

Future PK–PD frameworks should also incorporate microbiota-dependent metabolism and microbial metabolite kinetics, as conventional plasma-based pharmacokinetic models may inadequately capture the indirect biological activity of polysaccharides. Integration of host–microbiome systems pharmacology may therefore represent an important next step for accurately predicting therapeutic responses.

### 7.3 Microbiome-targeted therapeutic strategies

The interaction between *Gastrodia elata* polysaccharides (GEPs) and the gut microbiota represents one of the most promising directions for future therapeutic development. Increasing evidence suggests that the biological activity of polysaccharides depends heavily on microbial fermentation processes that generate bioactive metabolites capable of influencing systemic cardiovascular pathways. Future studies should therefore move beyond descriptive microbiota analyses and focus on identifying specific microbial taxa, metabolite signatures, and functional microbial pathways responsible for mediating cardiovascular protection.

Several bacterial genera may play important roles in polysaccharide fermentation and short-chain fatty acid (SCFA) production. Genera such as *Faecalibacterium, Roseburia, Akkermansia, Bifidobacterium, and Lactobacillus* have been associated with degradation of complex dietary polysaccharides and generation of metabolites involved in immune and metabolic regulation (Tang et al., 2019; Witkowski et al., 2020). In particular, SCFA-producing bacteria such as *Faecalibacterium prausnitzii* and *Roseburia* species may contribute to anti-inflammatory and endothelial-protective effects through modulation of immune signaling, intestinal barrier integrity, and vascular homeostasis.

Future microbiome-focused studies should integrate metagenomic sequencing, metabolomics, and microbial functional profiling to identify microbial signatures associated with favorable cardiovascular responses to GEP administration. Such approaches may help establish responder and non-responder phenotypes and improve understanding of inter-individual variability in therapeutic efficacy.

Importantly, microbiome-targeted therapeutic strategies may provide opportunities to enhance the biological activity of GEPs. Potential approaches include co-administration of probiotics capable of promoting polysaccharide fermentation, use of prebiotic formulations to selectively enrich SCFA-producing bacterial populations, and personalized dietary interventions designed to optimize gut microbial composition. In addition, fecal microbiota transplantation (FMT) models may serve as valuable experimental tools for establishing causal relationships between microbial composition and GEP-mediated cardiovascular effects. Transfer of microbiota from GEP-responsive models into germ-free or dysbiotic recipients could help clarify the contribution of specific microbial communities to observed therapeutic outcomes.

Another promising direction involves development of synbiotic formulations combining GEPs with selected probiotic strains to improve fermentation efficiency, metabolite generation, and therapeutic consistency. Such strategies may ultimately facilitate precision microbiome-based cardiovascular interventions tailored to individual microbial profiles and metabolic phenotypes. However, many of these microbiome-targeted strategies remain conceptual and require experimental validation in GEP-specific cardiovascular models.

### 7.4 Advanced drug delivery systems

One of the major barriers to clinical translation is the low bioavailability of GEPs. To address this limitation, advanced delivery strategies including nanoparticle-based carriers, liposomal formulations, and hydrogel-based systems should be further investigated to improve stability, controlled release, tissue targeting, intestinal absorption, and protection from enzymatic degradation (Peer et al., 2007). Such innovations could significantly enhance the therapeutic efficacy of GEPs. Future delivery systems may additionally be designed to selectively target intestinal microbial niches or regulate controlled release within specific regions of the gastrointestinal tract in order to optimize microbial fermentation efficiency and metabolite production.

### 7.5 Combination therapy and synergistic approaches

Given their multi-target biological properties, GEPs may have potential as adjunctive agents alongside established cardiovascular therapies. Future investigations should explore whether combination approaches involving lipid-lowering, antithrombotic, or anti-inflammatory treatments could enhance therapeutic efficacy while potentially reducing treatment burden and adverse effects associated with intensive pharmacotherapy.

### 7.6 Precision medicine and patient stratification

The heterogeneity of cardiovascular disease suggests that a “one-size-fits-all” therapeutic approach may be suboptimal. Future research should therefore explore the role of GEPs within precision medicine frameworks that incorporate genetic variability, metabolic profiling, and microbiome composition. Identification of patient subgroups most likely to benefit from GEP-based interventions could improve therapeutic outcomes and facilitate more individualized treatment strategies (Ashley, 2016).

Future precision medicine frameworks may also incorporate microbiome stratification approaches, enabling selection of patients with favorable microbial signatures associated with enhanced polysaccharide fermentation and SCFA generation.

### 7.7 Clinical trial design and outcome prioritization

To establish clinical relevance, there is an urgent need for well-designed randomized controlled trials evaluating GEPs in cardiovascular disease. Future studies should employ standardized and well-characterized GEP formulations, incorporate appropriate control groups and blinding procedures, focus on clinically meaningful cardiovascular outcomes, and include longer-term follow-up. In addition, integration of biomarker-driven endpoints may help elucidate mechanisms, improve pharmacodynamic assessment, and identify potential treatment responders (Califf, 2018; Strimbu and Tavel, 2010).

## 8 Clinical implications

From a clinical standpoint, GEPs may be more appropriately conceptualized as modulators of the cardiometabolic environment rather than as conventional pharmacological agents. Their potential role may lie in influencing interconnected biological processes such as inflammation, oxidative stress, and endothelial function through indirect and system-level mechanisms. The emerging evidence on *Gastrodia elata* polysaccharides (GEPs) suggests potential relevance in cardiovascular care; however, their current role is best viewed as adjunctive and exploratory rather than definitive. Translating preclinical promise into clinical utility requires careful interpretation within the context of existing evidence-based cardiovascular therapies.

### 8.1 Potential role as adjunctive therapy

Given their multi-target biological effects—particularly anti-inflammatory, antioxidant, and endothelial-protective actions—GEPs may have potential as adjunctive agents alongside standard cardiovascular treatments. In conditions such as atherosclerosis, hypertension, and metabolic syndrome, where residual inflammatory and oxidative risk persists despite optimal therapy, GEPs could theoretically provide complementary mechanistic benefits (Ridker, 2016; Libby et al., 2009). However, until robust clinical data are available, their use should not replace established therapies with proven mortality and morbidity benefits.

### 8.2 Target patient populations

From a clinical perspective, GEP-based interventions may be most relevant in cardiometabolic phenotypes characterized by persistent low-grade inflammation, oxidative stress, and endothelial dysfunction despite guideline-directed therapy, such as patients with metabolic syndrome, type 2 diabetes, and early atherosclerotic disease. In particular, individuals with residual inflammatory risk or endothelial dysfunction not fully addressed by lipid-lowering or antihypertensive therapies may represent a rational target population for adjunctive interventions. Additionally, the microbiota-mediated effects of GEPs suggest potential utility in patients with cardiometabolic dysregulation linked to gut microbiome alterations, although this remains an area for future clinical validation (Tang et al., 2017).

### 8.3 Integration with current therapeutic frameworks

Any potential clinical application of GEPs should be considered within the framework of evidence-based cardiovascular management, including lipid-lowering therapy, antihypertensive treatment, antithrombotic strategies, and lifestyle modification (Visseren et al., 2021). At present, GEPs are more appropriately conceptualized as nutraceutical or functional adjuncts rather than primary pharmacological therapies, with potential utility in combination strategies for patients who demonstrate incomplete response to standard treatments. Nevertheless, clinicians should remain cautious regarding possible herb–drug interactions, particularly in patients receiving antiplatelet or anticoagulant therapy.

### 8.4 Safety and tolerability considerations

Although plant-derived polysaccharides are generally considered biologically safe, clinical safety data specific to GEPs remain limited. Important concerns include variability in product composition, lack of standardized dosing strategies, and the potential for interactions with conventional cardiovascular medications. Until more robust safety evidence becomes available, GEP use should be approached with clinical caution, particularly in elderly individuals and patients with multiple comorbidities or polypharmacy exposure (Posadzki et al., 2020).

### 8.5 Implications for clinical research

The current evidence highlights an urgent need for well-designed clinical trials to more clearly define the therapeutic role of GEPs in cardiovascular care. Future investigations should evaluate standardized and well-characterized GEP formulations in adequately powered patient populations while focusing on clinically meaningful cardiovascular outcomes rather than surrogate biomarkers alone. Integration of mechanistic and biomarker-driven endpoints may further improve understanding of pharmacodynamic responses, treatment heterogeneity, and potential responder populations. Such studies will be essential to determine whether the promising biological effects observed in preclinical models can ultimately translate into meaningful clinical benefit.

## 9 Conclusion

In summary, *Gastrodia elata* polysaccharides represent a biologically active yet mechanistically complex class of compounds with potential relevance in cardiovascular disease. The coexistence of limited systemic bioavailability and significant experimental efficacy highlights a bioavailability–efficacy paradox that challenges traditional pharmacological interpretations. A shift toward a microbiota-centered and systems-oriented framework provides a more coherent explanation for their observed effects and offers a foundation for future translational research. Advancing this field will require rigorous mechanistic validation, standardized characterization of polysaccharide preparations, and well-designed clinical studies to determine their true therapeutic value. The present review should therefore be interpreted within the context of a still-developing evidence base dominated by preclinical and hypothesis-generating studies.
